# Phase Precession Relative to Turning Angle in Theta‐Modulated Head Direction Cells

**DOI:** 10.1002/hipo.70008

**Published:** 2025-03-12

**Authors:** Zilong Ji, Eleonora Lomi, Kate Jeffery, Anna S. Mitchell, Neil Burgess

**Affiliations:** ^1^ UCL Institute of Cognitive Neuroscience, University College London London UK; ^2^ UCL Queen Square Institute of Neurology, University College London London UK; ^3^ Department of Neuroscience, Physiology and Pharmacology University College London London UK; ^4^ School of Psychology & Neuroscience, University of Glasgow Glasgow UK; ^5^ School of Psychology, Speech, and Hearing, University of Canterbury Christchurch New Zealand

**Keywords:** firing rate adaptation, head direction cells, ring attractor network, theta modulation, theta phase precession, theta skipping

## Abstract

Grid and place cells typically fire at progressively earlier phases within each cycle of the theta rhythm as rodents run across their firing fields, a phenomenon known as theta phase precession. Here, we report theta phase precession relative to turning angle in theta‐modulated head direction cells within the anteroventral thalamic nucleus (AVN). As rodents turn their heads, these cells fire at progressively earlier phases as head direction sweeps over their preferred tuning direction. The degree of phase precession increases with angular head velocity. Moreover, phase precession is more pronounced in those theta‐modulated head direction cells that exhibit theta skipping, with a stronger theta‐skipping effect correlating with a higher degree of phase precession. These findings are consistent with a ring attractor model that integrates external theta input with internal firing rate adaptation—a phenomenon we identified in head direction cells within AVN. Our results broaden the range of information known to be subject to neural phase coding and enrich our understanding of the neural dynamics supporting spatial orientation and navigation.

## 1 Introduction

There are many examples of neurons that encode information via changes in their firing rates. One of the most striking of these signals is provided by head direction cells (HDC) recorded in freely moving rodents (Taube et al. 1990). Each HDC fires at a high rate whenever the animal's head is oriented in the cell's “preferred direction” so that a population of HDCs with different preferred directions signals the animal's current head direction to downstream areas. Similarly, place cells fire at a high rate whenever the animal enters a preferred environmental location, so that an active population of place cells, with overlapping preferred locations signals the animal's current environmental location.

An additional coding mechanism would be a phase code, in which information is encoded by the phase of an ongoing oscillation at which a neuron fires spikes (rather than the rate at which it fires them). The ongoing oscillation of relevance to the present study is the theta rhythm, which in rats is a large‐amplitude 6–11‐Hz oscillation in the local field potential (LFP), seen in the hippocampus and related structures when the animals are locomoting (Vanderwolf 1969). This arises from synchronous phasic multi‐unit activity. Many neurons, including place cells, are burst‐modulated at (or near) the theta frequency, and some neurons show an effect called “theta‐skipping” in which they only fire on every alternate theta cycle (Brandon et al. 2013; Lomi et al. 2023), for reasons we explore here.

Theta rhythm provides a clock‐like reference against which neural activity can be characterized according to the phase at which spikes occur. One of the most robust examples of phase coding is seen in the “theta phase precession” of place cell firing (O'Keefe and Recce 1993), in which the cell fires at successively earlier phases of the theta rhythm as the animal moves through the firing field. This phase code provides additional information about the animal's location beyond that encoded in firing rates (Jensen and Lisman 2000). A consequence of the theta phase precession of individual place cells is that, within each theta cycle, the sub‐population of active place cells sweeps forward from those with firing fields peaked behind the animal (firing at an early phase, later in the precession process) to those with fields peaked ahead of the animal (firing at late phases, that have just started to precess) (Burgess et al. 1992; Skaggs et al. 1996). This phenomenon, called “theta sweeps,” indicates direction of movement, beyond what is conveyed by firing rates (Johnson and Redish 2007; Bush and Burgess 2020). It also rehearses the recent and upcoming sequence of cell firing, which may be useful in spatial memory and navigation. Similar phenomena are seen in grid cells in the medial entorhinal cortex (Hafting et al. 2008) and outside the hippocampal formation (Jones and Wilson 2005; Van Der Meer and Redish 2011), as well as in hippocampal phase precession of non‐spatial information (Aronov et al. 2017; Terada et al. 2017). However, robust examples of phase coding relative to other spatial dimensions outside of the hippocampal formation are rare (Siegel et al. 2009).

The classic HDCs are found in a circuit including the dorsal tegmental nuclei of Gudden, lateral mammillary nuclei, anterodorsal thalamic nucleus (ADN), dorsal presubiculum, and retrosplenial cortex (Taube 2007). These fire at a high rate when the head faces in the cell's preferred firing direction, do not show firing rate adaptation if the head remains there, and do not show modulation by theta oscillations. However, a parallel circuit exists, incorporating the ventral tegmental nucleus of Gudden, medial mammillary nuclei, anteroventral thalamic nucleus (AVN), and retrosplenial cortex (Aggleton et al. 2010; Lomi et al. 2021), within which theta‐modulated HDCs have been reported in AVN (Tsanov et al. 2011), particularly in the ventrolateral subfield (Lomi et al. 2023). A proportion of these theta‐modulated head direction cells (tmHDCs) show an intrinsic firing frequency that exceeds that of the LFP theta rhythm—a potential indicator of theta phase coding (Lomi et al. 2023). However, neither phase precession, theta skipping, nor firing rate adaptation has been explicitly looked for here. For theoretical reasons deriving from modeling, described later, we decided to re‐analyze a previously collected data set (Lomi et al. 2023) to search for these phenomena.

Specifically, we investigate the possibility that tmHDCs show theta phase coding of the angle turned, in an analogous way to the theta phase coding of distance traveled through the firing fields shown by place cells (O'Keefe and Recce 1993) and by a proportion of grid cells (Hafting et al. 2008). On finding this, we compare our findings to a continuous attractor model of tmHDCs in which theta phase precession emerges due to external theta input and internal firing rate adaptation in the same way that it does for place cells (Chu et al. 2024). Importantly, theta skipping also emerges from this model. Further analysis of the data revealed both theta skipping and firing rate adaptation under specific, model‐predicted conditions. Our model thus connects seemingly distinct single‐cell firing features in HDCs—including theta phase coding, theta skipping, and firing rate adaptation—to various aspects of population dynamics within the head direction network, providing a unified framework for understanding information coding in spatial orientation and navigation.

## 2 Materials and Methods

### 2.1 Animals

Single unit data were part of a previously published experiment (Lomi et al. (2023)). Full details of animals, surgical, and recording procedures are reported in the original paper. Raw data and analysis results are available in the corresponding data repository (https://figshare.com/s/d2e540c7af8d308848f3). Experiments were carried out in accordance with the UK Animals (Scientific Procedures) Act 1986 and EU Directive (2010‐63‐EU), complying with ARRIVE guidelines for the care and use of laboratory animals.

### 2.2 Surgery

Standard stereotaxic surgery techniques for implantation of chronic recording electrodes in behaving animals were followed (Szymusiak and Nitz 2002). Implant coordinates were as follows: AP, −1.7; ML, ± 1.4–1.7; DV, 3.4–3.6 (in mm from Bregma).

### 2.3 Recording Protocol and Testing Apparatus

Animals were screened daily for single‐unit activity. When a set of units was isolated, the experimental session was conducted. Screening and experimental sessions took place inside a 90 × 90‐cm open field square arena with 60‐cm high walls, within a cue‐rich room. The experimental session consisted of two 16‐min‐long trials, during which animals foraged for rice scattered inside the arena. At the end of each screening and experimental session, animals were removed from the arena and transferred back to the home cage, and the arena floor was cleaned before the beginning of a new session.

### 2.4 Intrinsic Theta Frequency

To quantify the frequency of theta modulation in individual HDCs, we applied the method proposed by Royer et al. (2010). First, we calculated the autocorrelogram of each cell's spike train, using 10‐ms bins from −500 to +500 ms, normalized to its maximum value, and smoothed with a 20‐bin boxcar. We then fitted a cosine wave function with frequency, to the autocorrelogram:

(1) $$y\left(t\right)=\left[a\left(\cos\left(2\mathrm{\pi \omega t}\right)+1\right)+b\right]\cdot \exp\left(-\frac{\left|t\right|}{\tau_{1}}\right)+c\cdot \exp\left(-\frac{t^{2}}{\tau_{2}^{2}}\right),$$

where t represents the time lag of the autocorrelogram (in seconds), $a,b,c,\tau_{1},\tau_{2}$ are the parameters determined by fitting. These parameters were constrained within the following range: $a\in \left[0,100\right],b\in \left[0,100\;\right],c\in \left[0,0.8\right],\omega \in \left[6,12\right],\tau_{1}\in \left[0,8\right]$ and $\tau_{2}\in \left[0,0.05\right]$. The first Gaussian term, $\exp\;\left(-|t|/\tau_{1}\right)$, was used to fit the decay of the autocorrelogram's amplitude with increasing lag, while the second Gaussian term, $c\cdot \exp\;\left(-t^{2}/\tau_{2}^{2}\;\right)$, was used to fit the central peak of the autocorrelogram.

### 2.5 Theta‐Skipping Index

To quantify the theta‐skipping effect in HDCs, we applied the method from Brandon et al. (2013), fitting the autocorrelogram with a cosine wave of frequency ω and an interfering cosine wave of frequency ω/2:

(2) $$\begin{array}{c} \begin{array}{c} y\left(t\right)=\left[a_{1}(\cos\left(2\mathrm{\pi \omega t}\right)+1\right)+a_{2}\left(\cos\left(\mathrm{\pi \omega t}\right)+1\right)+b]\cdot \exp\left(-\frac{\left|t\right|}{\tau_{1}}\right)\\ \;+c\cdot \exp\left(-\frac{t^{2}}{\tau_{2}^{2}}\right), \end{array} \end{array}$$

where t is the time lag of the autocorrelogram (in seconds), and $a_{1},a_{2},b,c,\tau_{1},\tau_{2}$ are the parameters determined by fitting. These parameters were constrained within the following range: $a_{1}\in \left[0,100\right],a_{2}\in \left[0,100\right],b\in \left[0,100\;\right],c\in \left[0,0.8\right],\omega \in \left[6,12\right],\tau_{1}\in \left[0,8\right]$ and $\tau_{2}\in \left[0,0.05\right]$. The interfering cosine wave was used to capture the theta‐skipping effect on the autocorrelogram. The theta‐skipping index was then calculated as the difference between the first and second peaks on the fitted curve, normalized by the larger of the two:

(3) $$\begin{array}{c} \mathrm{TS}=\frac{p_{2}-p_{1}}{\max\;\left(p_{1},p_{2}\right)}, \end{array}$$

where $p_{1}$ is the model value at one cycle with t=2π/ω and $p_{2}$ is the model value at two cycles with t=4π/ω. This index is bounded between −1 and 1, with higher values indicating a greater degree of theta skipping. The TS index was calculated using all the spikes and after filtering for spikes emitted during periods of non‐rotations.

### 2.6 Classification of HDCs

To quantify the degree of directional tuning of a recorded cell, the cell's firing rates were binned into 60 discrete angular bins (6° each) and smoothed with a 5‐bin (30°) smoothing kernel. The Rayleigh vector length was then calculated as

(4) $$\begin{array}{c} R=\frac{1}{n}\sqrt{{\left(\sum_{i}f_{i}\cos\left(\theta_{i}\right)\right)}^{2}+{\left(\sum_{i}f_{i}\sin\left(\theta_{i}\right)\right)}^{2}}, \end{array}$$

where $f_{i}$ is the firing rate in the *i*th angular bin, $\theta_{i}$ is the direction (in radians) of the *i*th bin, and n is the total number of bins. Cells were classified as HDCs if (1) the R‐vector passes the 99th percentile shuffle cutoff and (2) the peak firing rate in the directional field is more than 1 Hz.

Additionally, each HD cell was classified as theta‐modulated based on two criteria. The first criterion was the index of rhythmicity (IR), calculated as the difference between the theta modulation trough (autocorrelogram value between 60 and 70 ms) and the theta modulation peak (autocorrelogram value between 120 and 120 ms), divided by their sum. The index is bounded between −1 and 1. The second criterion was the index of theta phase‐coupling (IC), representing the phase of theta at which spikes occurred. A HD cell was considered theta‐modulated if it passed the 99th percentile shuffle cutoff for IC and had an IR ≥ 0.001.

Finally, a theta‐modulated HD cell was classified as a theta‐skipping cell if (1) it showed a good fit (R2 > 0.7) of the model parameters to the autocorrelogram in Equation (2); (2) the theta‐skipping index TS was greater than 0.1. As reported in Lomi et al. (2023), all classifications were confirmed by visual inspection of each cell's autocorrelogram.

### 2.7 Phase Precession Relative to Turning Angle

Importantly, only periods of continuous head rotation were selected for phase precession analysis. We quantified phase precession in tmHDCs relative to “turning angle,” which is how far through a cell's tuning curve the head direction had progressed during a head turn. Specifically, to calculate the turning angle, we first smoothed angular speed using a Gaussian filter with a 0.4‐s standard deviation, then selected periods with a minimum angular speed of 0.5 rad per second and a duration of at least 0.5 s. This results in at least 15° of continuous head rotation.

The trial‐averaged preferred firing direction of a cell was taken as the zero point. We plotted the spike phase against the turning angle for each cell. Spike phases were computed by applying the Hilbert transform to the bandpass‐filtered LFP signal in the theta frequency range (6–12 Hz).

In phase precession studies of place cells when animals run on linear tracks, cells often develop directional tuning; thus, only unidirectional running periods are typically analyzed. However, HD cells fire during both clockwise and counterclockwise turns within their tuning fields. To account for directional differences in phase precession, we flipped the head directions during counterclockwise turns by subtracting the angle from 2π. This adjustment ensures that small values on the x‐axis consistently represent entry phases into the tuning field, while large values represent exit phases.

Finally, we performed circular–linear correlation analysis (Kempter et al. 2012) for each cell. Cells were considered to show significant phase precession relative to turning angle if (1) the *p* value was less than 0.05; (2) the correlation coefficient was negative.

### 2.8 The Computational Model

tmHDCs were modeled using a ring attractor network with the following form:

(5) $$\begin{array}{c} \tau \frac{\partial r_{i}}{\partial t}=-r_{i}+\sum_{j=1}^{N}J_{\mathrm{ij}}f\left(r_{j}\right)-a_{i}+O_{M}\left(v\right)I_{i}\left(\theta \right), \end{array}$$

where $r_{i}$ is the pre‐synaptic input to the *i*th cell and N is the number of cells evenly distributed on the ring. Each cell receives four signals: (1) head direction‐dependent sensory input, $I_{i}^{h}\left(\theta \right)$; (2) theta oscillatory input from the medial septum and/or ventral tegmental nuclei of Gudden, $O_{M}\left(v\right)$; (3) recurrent input from other cells; and (4) firing rate adaptation, $a_{i}$.

First, the directional input is modeled as a Gaussian input:

(6) $$\begin{array}{c} I_{i}\left(\theta \right)=A\exp\left[\frac{{\left(x_{i}-\theta \right)}^{2}}{4b^{2}}\right], \end{array}$$

where A represents the input strength, $x_{i}$ is the preferred firing direction of the ith neuron on the ring attractor, b controls the width of the Gaussian input, and θ is the animal's current head direction.

Second, theta modulation is modeled as a sinusoidal wave:

(7) $$\begin{array}{c} O_{M}\left(v\right)=1+\mathrm{av}\sin\;\left(2\mathrm{\pi \omega t}\right), \end{array}$$

where ω is the oscillation frequency. Theta modulation amplitude scales linearly with the angular speed v, where α is the scaling factor, that is, as the animal turns faster, theta modulation becomes stronger.

Third, the recurrent connections are modeled with a translationally invariant Gaussian function:

(8) $$\begin{array}{c} J_{\mathrm{ij}}=\frac{J_{0}}{2\pi b^{2}}\exp\left[\frac{{\left\|\phi_{i}-\phi_{j}\right\|}^{2}}{4b^{2}}\right], \end{array}$$

where $\left\|\cdot \right\|$ denotes the circular distance, $J_{0}$ represents the connection strength, and b controls the width of directional tuning. The translationally invariant form implies that synaptic connection strength between two HD cells depends only on the relative distance of their preferred directions on the ring.

The firing rate of the *j*th cell, $f\left(r_{j}\right)$ is modeled with a global inhibition function, expressed as

(9) $$\begin{array}{c} f\left(r_{j}\right)=\frac{r_{j}^{2}}{1+k\sum_{j}r_{j}^{2}}, \end{array}$$

where k represents the global inhibition strength. Global inhibition is essential for maintaining a localized activity bump on the ring attractor.

Lastly, cells in the head direction attractor network exhibit firing rate adaptation, modeled as a negative feedback inhibition to reduce neural firing:

(10) $$\begin{array}{c} \tau_{a}\frac{\partial a_{i}}{\partial t}=-a_{i}+\mathrm{mf}\left(r_{i}\right), \end{array}$$

where $\tau_{a}$ is the time constant of firing rate adaptation, with $\tau_{a}\gg \tau$, indicating that firing rate adaptation operates on a slower timescale than cell firing. The parameter m represents adaptation strength. At the single‐neuron level, firing rate adaptation reduces firing frequency following an initial increase in response to an input of constant intensity.

At the network level, firing rate adaptation induces intrinsic mobility of the activity bump (Mi et al. 2014), a key mechanism for introducing activity sweeps in continuous attractor network models of place cells (see also Hopfield (2010)). In those models, firing rate adaptation causes the sub‐population of active place cells to sweep forward from those with firing fields behind the animal (as adaptation reduces their firing rates) to those with firing fields ahead of the animal, within each theta cycle. This corresponds to theta phase precession within individual place cells that fire at late phases when their fields are ahead of the animal and at earlier phases when their fields move behind the animal (Burgess et al. 1992; Skaggs et al. 1996).

## 3 Results

### 3.1 Three Types of HDCs

**FIGURE 1:**
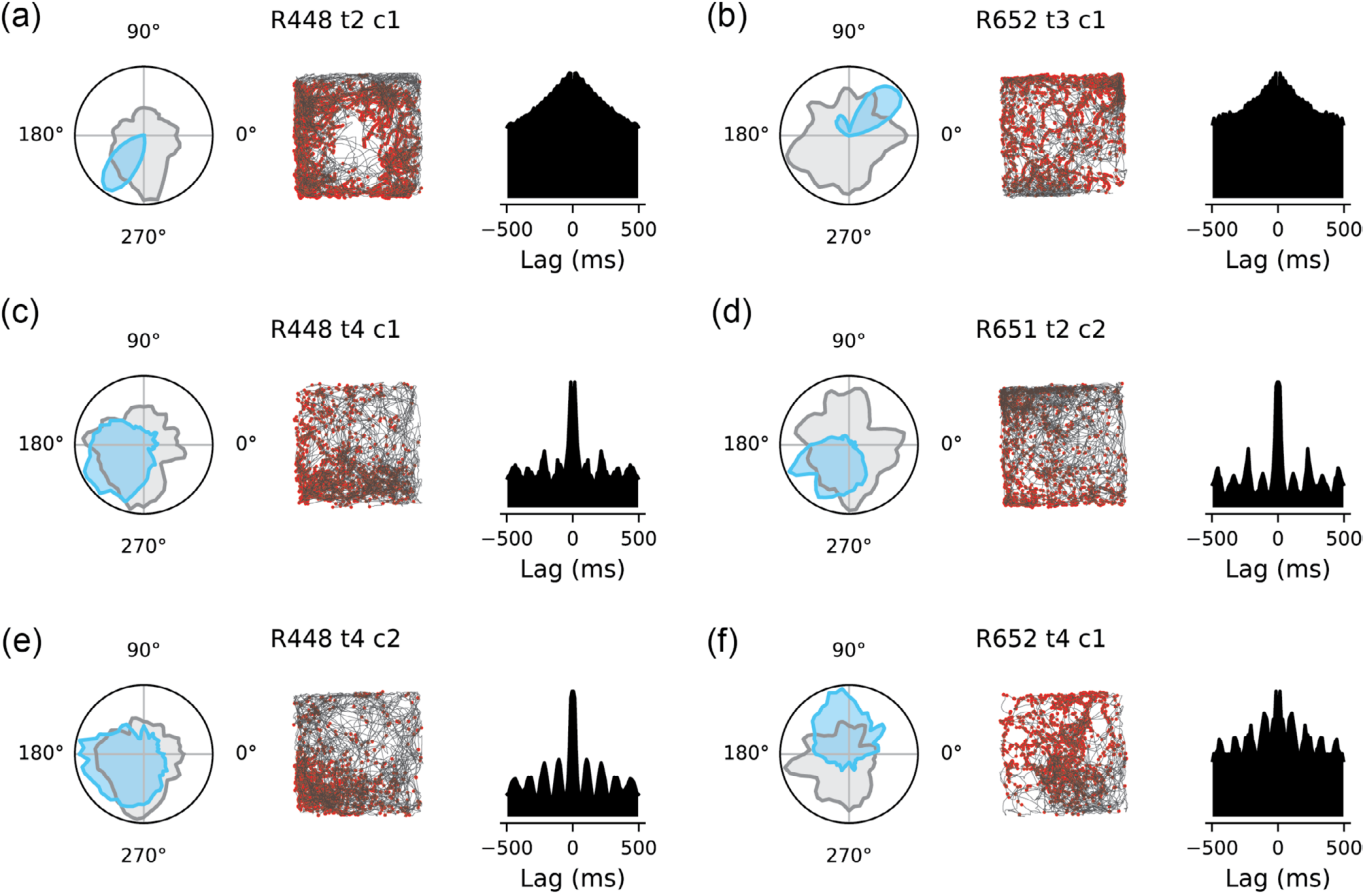
Examples of three types of head direction cells (HDCs) in the anteroventral thalamic nucleus. In each panel, left: The directional tuning curve of a HDC (blue) and the dwell time in each directional bin (gray); middle: The spike map (red dots) of the cell with running trajectory marked in gray; right: The autocorrelogram of the spike train of the cell. The name for a cell was shown at the top of each panel. (a, b) Two example classic HDCs; (c, d) two example theta‐modulated head direction cells (tmHDCs) with the theta‐skipping effect; and (e, f) two example tmHDCs without the theta‐skipping effect.

Previous studies have shown that HDCs in the AVN can be divided into two groups: non‐theta‐modulated (classic HDCs; Figure 1a,b; 136/359) and theta‐modulated HD cells (tmHDCs; Figure 1c–f; 223/359) (Tsanov et al. 2011; Lomi et al. 2023). tmHDCs can be further classified based on their spike autocorrelograms: one group exhibits a theta‐skipping effect (Methods; Figure 1c,d), firing on alternate theta cycles (theta‐skipping HDCs; 75/223), while the other group does not display this effect (theta non‐skipping HDCs; Figure 1e,f; 148/223).

Overall, tmHDCs exhibited less‐precise directional tuning than classic HDCs, with both lower R‐vector values (0.26±0.18 vs. 0.44±0.26; *p* = $2.5\times 10^{-11}$) and broader tuning widths (137.8±19.6 vs. 117.1±32.2; *p* = $2.5\times 10^{-11}$). tmHDCs also showed relatively lower peak firing rates than classic HDCs (7.4±10.6 vs. 10.3±18.0), although this difference was not statistically significant (*p* = 0.207).

### 3.2 Phase Precession Relative to Turning Angle in tmHDCs

**FIGURE 2:**
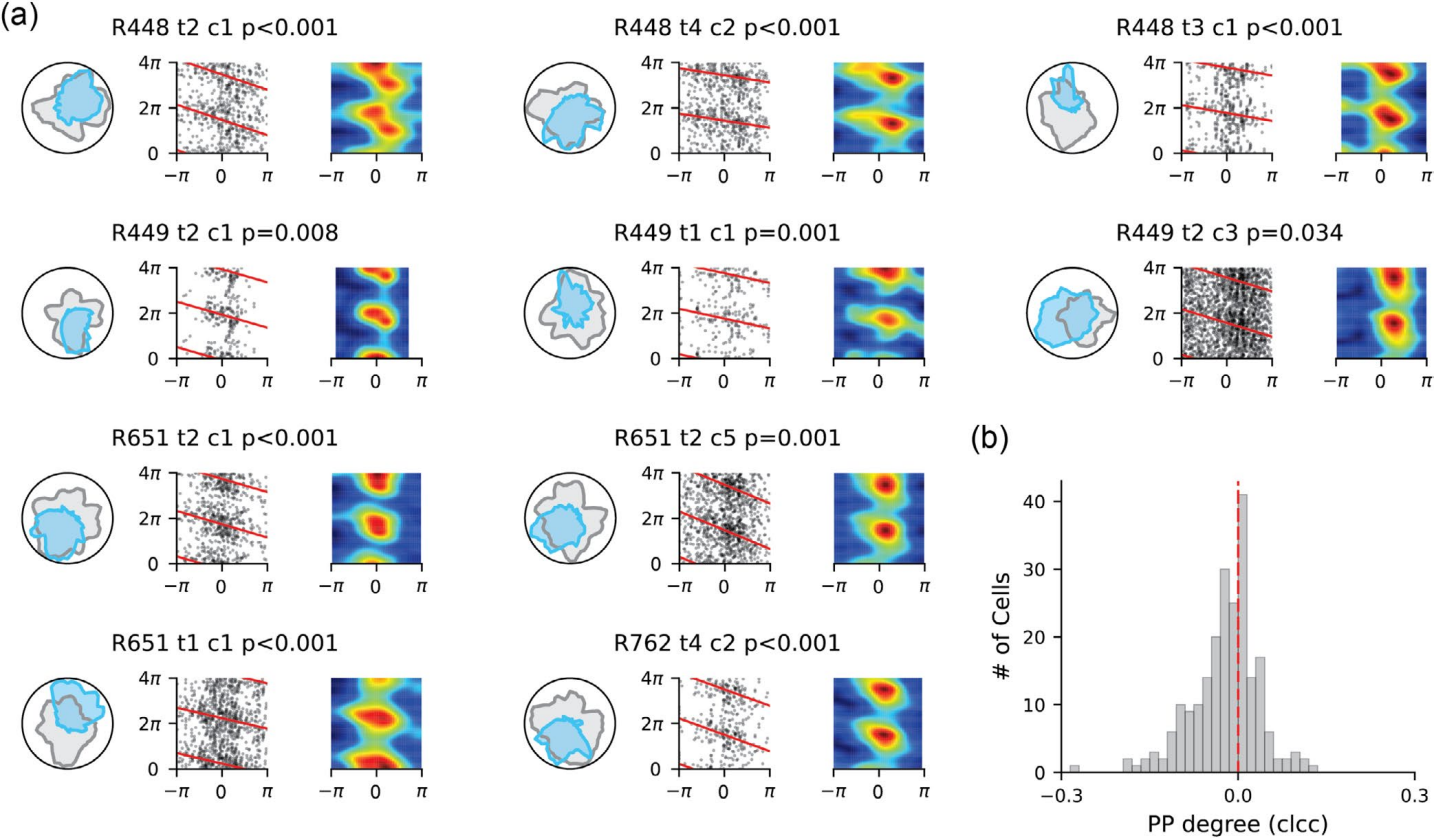
Examples of theta‐modulated head direction cells (tmHDCs) showing theta phase precession relative to turning angle. (a) In each panel, left: The tuning field of a tmHDC (blue) and the dwell time in each directional bin (gray); middle: Spike phase against the turning angle in the directional tuning curve, with the preferred firing direction centered at 0° and red lines representing the circular–linear fit; right: 2D heat map plot of spike phase against turning angle. The cell name and the *p* value of circular–linear correlation are shown at the top of each panel. (b) The histogram of circular–linear correlation coefficients (clcc) of spike phase against angle from all identified tmHDCs (223 in total), with the red line representing a correlation coefficient of zero.

Grid and place cells have been shown to exhibit theta phase precession when animals traverse their firing fields (O'Keefe and Recce 1993; Hafting et al. 2008). Beyond location‐based phase coding, hippocampal CA1 cells have also been reported to phase‐code non‐spatial event sequences (Aronov et al. 2017; Terada et al. 2017). Here we further demonstrate phase precession relative to turning angle in tmHDCs. Specifically, as the animal rotates its head, these cells fire at progressively earlier theta phases as the head direction sweeps through their directional firing fields (Figure 2a). Phase coding was quantified using a circular–linear correlation (Methods). Across all identified tmHDCs (223 in total), the correlation coefficients were significantly negative (Figure 2b; one‐sample *t*‐test with t(222) = −5.60, *p* = $6.22\times 10^{-8}$), indicating robust phase precession as the head turned through their directional firing fields. Overall, 35 HDCs (15.7%; 35/223) exhibited significant phase precession (*p* < 0.05 and negative correlation coefficients). We return later to the question of what happens if the head remains stably oriented within the cell's preferred firing direction.

**FIGURE 3:**
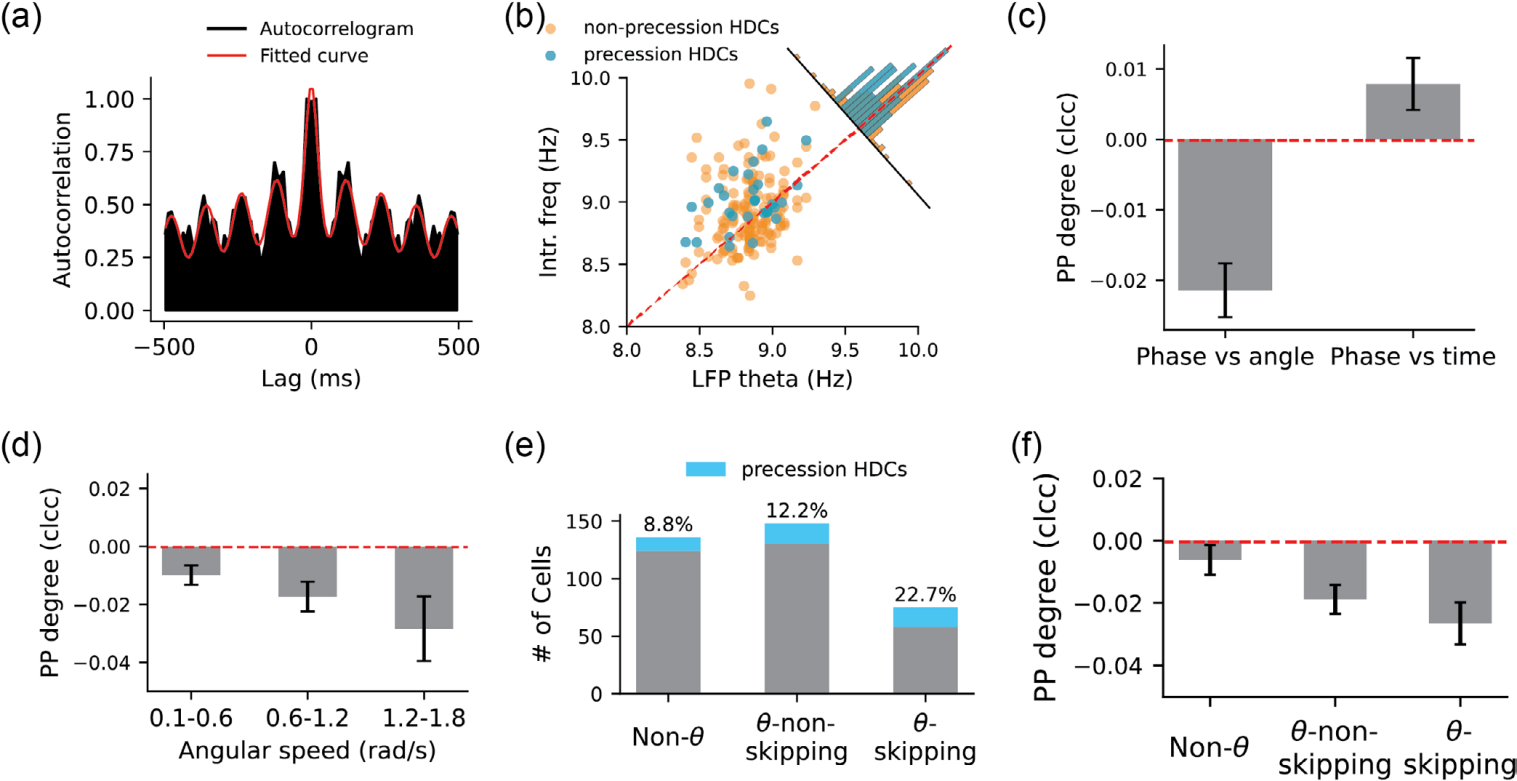
Features of theta phase precession relative to turning angle in theta‐modulated head direction cells (tmHDCs). (a) The temporal autocorrelogram (between ±500 ms) of the spike train of a tmHDC (dark) and the fitted curve (red; see Methods). (b) The intrinsic firing frequency against LFP theta rhythm of all tmHDCs. Blue dots mark tmHDCs exhibiting significant phase precession relative to turning angle and orange dots mark tmHDC not exhibiting significant phase precession. The red dashed line marks the diagonal line and corner histograms display the distance of each marker from the diagonal. (c) The degree of phase precession of all tmHDCs (measured by the circular–linear correlation coefficient; [CLCC]) as a function of turning angle and time since turning, with bars representing the standard error of the mean CLCC. (d) The degree of phase precession of all tmHDCs as a function of angular head velocity. (e) Percentage of HDCs in three types of significant theta phase precession relative to turning angle. (f) Averaged degree of phase precession relative to turning angle in three types of HDCs.

When an animal traverses a linear track, a phase‐precessing place cell exhibits an intrinsic firing frequency higher than the LFP theta frequency (O'Keefe and Recce 1993; Geisler et al. 2007). Therefore, if a HDC exhibits phase precession relative to turning angle, its intrinsic theta‐burst frequency should likewise exceed the LFP theta frequency. To test this, we calculated each HDC's intrinsic firing frequency (Methods) and found that HDCs with significant phase precession relative to turning angle exhibited higher intrinsic theta‐firing frequencies than those without (Figure 3a,b; Mann–Whitney U test with *p* = 0.033). It is also noteworthy that higher intrinsic theta‐firing frequency does not necessarily produce phase precession, for example, if the firing phase of the cell when the animal enters the tuning field is not constant over time, the intrinsic firing frequency can still be higher than LFP theta frequency without producing a stable relationship between firing phase and progress through the firing field (Feng et al. 2015).

Similar to place cells, where spike phase correlates more with position within the firing field than with time (O'Keefe and Recce 1993; Huxter et al. 2003), the spike phase of a tmHDC correlates more with turning angle in the directional tuning curve than with time since turning (Figure 3c; median circular–linear correlation coefficient [CLCC] for phase‐angle: −0.013; median CLCC for phase‐time: 0.007; Mann–Whitney U test with *p* = $1.7\times 10^{-7}$). Additionally, in all tmHDCs (223/359), the degree of phase precession increases as the animal turns faster through the directional tuning field (Figure 3d), closely aligning with the greater anticipatory coding with increased angular head velocity of HDCs in the anterior thalamic nucleus (Blair and Sharp 1995; Taube and Muller 1998).

### 3.3 Theta‐Skipping HDCs Have a Higher Probability of Showing Phase Precession

In place cells, single‐cell theta phase precession and populational theta sweeps reflect the same neural process, at least after the very first experience of an environment (Feng et al. 2015). Similarly, in tmHDCs, phase precession relative to turning angle and theta sweeps of internal direction should be interrelated. A recent study showed that the internal direction decoded from HDC population activity in the parasubiculum alternates across successive theta cycles from side to side of the head axis as animals move along a straight trajectory (Vollan et al. 2024), leading to a theta‐skipping effect at the single‐neuron level (Brandon et al. 2013; Lomi et al. 2023). We hypothesized that theta skipping and phase precession are interrelated in tmHDCs.

We conducted a chi‐square test to determine whether the proportion of cells showing phase precession differed across classic HDCs (8.8%;12/136), theta non‐skipping HDCs (12.2%;18/148) and theta‐skipping HDCs (22.7%;17/75). The test revealed a statistically significant difference between the groups (Figure 3e; $\chi^{2}\left(2\right)=8.33,p=0.016$). Post hoc pairwise comparisons using *z*‐tests with Bonferroni correction showed that the proportion of theta‐skipping HDCs exhibiting phase precession was significantly higher than that of classic HDCs (*z* = 2.80, *p* = 0.016). The differences between theta non‐skipping HDCs and classic HDCs (*z* = 0.91, *p* = 1.00) were not statistically significant. However, there was a trend toward a difference between theta‐skipping HDCs and theta non‐skipping HDCs (*z* = 2.04, *p* = 0.125), with the former exhibiting a higher proportion of cells showing phase precession (22.7% vs. 12.2%). This variation in the proportion of phase‐precessing cells across the three cell types was further reflected by the increasing degree of phase precession among them (Figure 3f).

It is noteworthy that classic HDCs, defined as those that did not meet the criteria for identifying tmHDCs (Methods), might still exhibit slight modulation by the LFP theta rhythm. Therefore, a small subset of these classic HDCs still demonstrated significant phase precession (8.8%). However, this proportion does not significantly differ from chance at the 5% significance level (one‐sample proportion *z*‐test: *z* = 1.57, *p* = 0.116).

**FIGURE 4:**
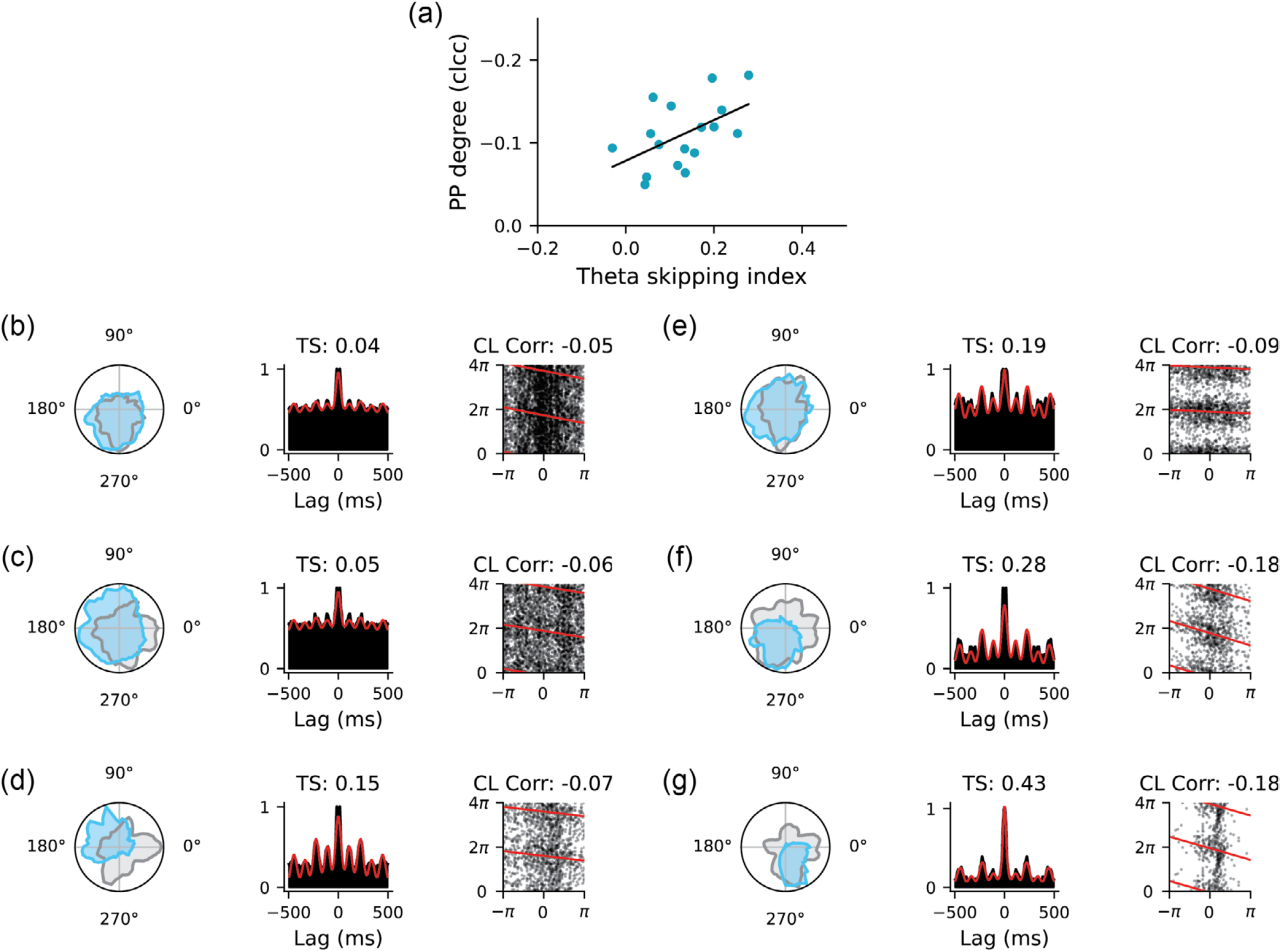
Relationship between the degree of theta skipping and the degree of phase precession relative to turning angle. (a) Degree of phase precession against theta‐skipping index for all theta‐modulated head direction cells (tmHDCs) with theta‐skipping effect. Note that the y axis was inversed. The dark line represents a linear fit. (b–g) Nine examples of tmHDCs with theta‐skipping effect with increased theta‐skipping index and degree of phase precession. In each panel, left: The tuning field of a cell (blue) and the dwell time in each directional bin (gray); middle: The temporal autocorrelogram (dark) and the fitted curve (red); right: Spike phase against the turning angle in the directional firing field, with the preferred firing direction centered at 0° and red lines representing the circular–linear fit.

Interestingly, for theta‐skipping HDCs exhibiting significant phase precession, the degree of phase precession significantly correlates with the theta‐skipping index (Pearson correlation, *r* = 0.52, *p* = 0.034; Figure 4). This result aligns with predictions from our computational model (see below).

### 3.4 Phase Coding Relative to Turning Angle Is Stable in Cells Recorded Across Trials

**FIGURE 5:**
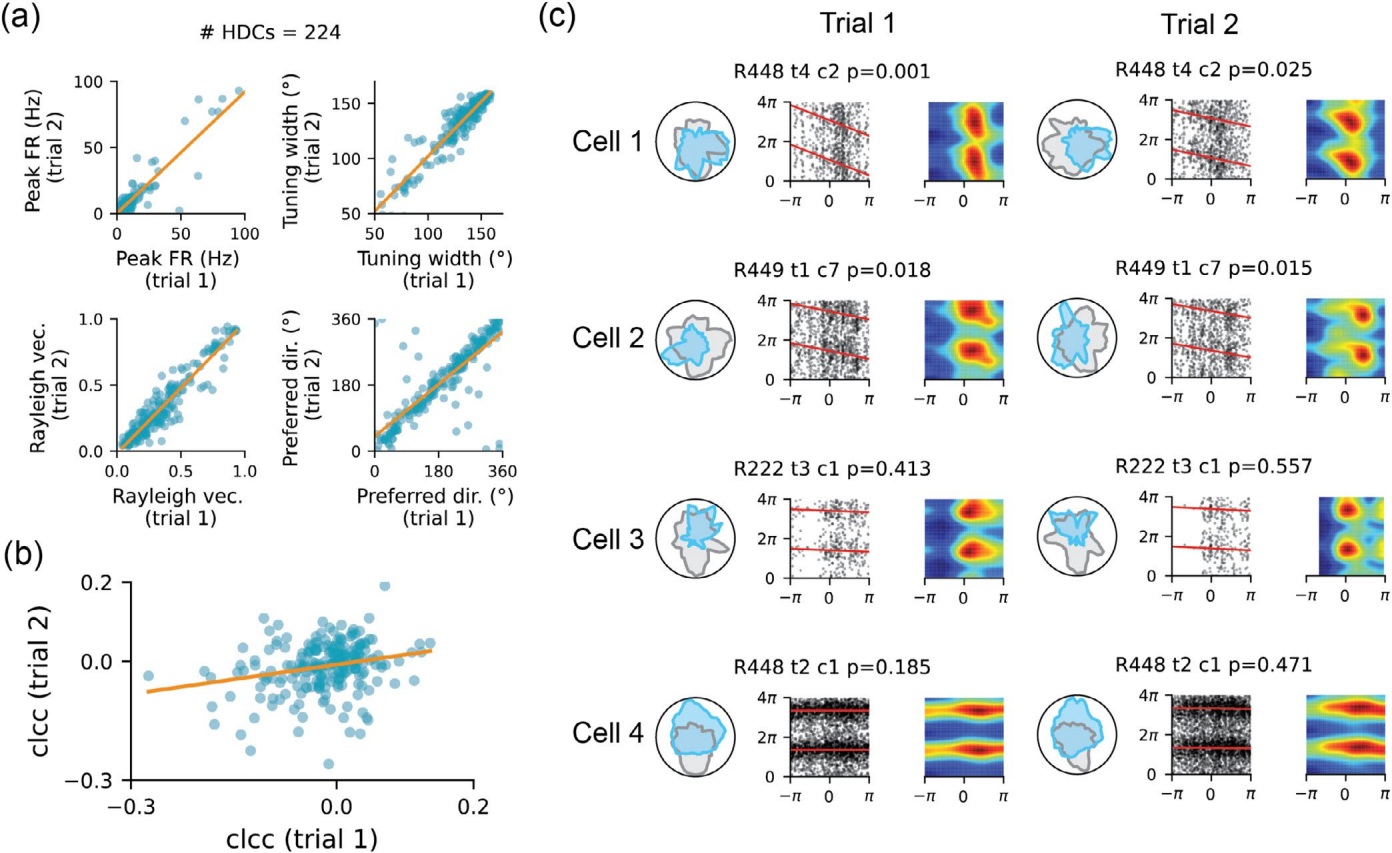
Phase coding stability in HDCs recorded across trials. (a) Firing characteristics of HDCs, including peak firing rates, directional tuning width, Rayleigh vector length, and preferred firing directions, demonstrate stability across two 16‐min trials. Each point represents an individual HDC, with orange lines indicating linear regression fits. (b) Circular–linear correlation coefficients exhibit a positive correlation between two recorded trials. (c) Four example HDCs recorded across two 16‐min trials. Cells 1 and 2 exhibit significant phase precession, while cells 3 and 4 do not display this property.

Since each recording session consisted of two 16‐min trials, we next examined whether the phase coding of turning angles in HDCs remained stable across the two trials. Across all recording sessions in six rats, we identified a total of 224 HDCs across the two trials. Key firing characteristics, including peak firing rate, directional tuning width, Rayleigh vector length, and preferred firing direction, were consistent for each cell across the two trials (Figure 5a). We then calculated the circular–linear correlation coefficients for each cell in both trials and examined their relationship across all 224 HDCs. A positive correlation was observed (Figure 5b; Pearson correlation with *r* = 0.223, *p* = $7.5\times 10^{-4}$), indicating that phase precession was a stable feature. For instance, cells showing phase precession in trial 1 also displayed phase precession in trial 2 (e.g., cells 1 and 2 in Figure 5c), while cells without phase precession in trial 1 tended to lack phase precession in trial 2 (e.g., cells 3 and 4 in Figure 5c).

It is worth noting that the relationship between circular–linear correlation coefficients was less tightly aligned along the diagonal compared to simpler firing features such as tuning width and preferred firing direction. This discrepancy may be due to (1) the higher‐order nonlinearity involved in calculating circular–linear correlation coefficients compared to straightforward firing features and (2) variations in behavioral differences such as the time spent facing the preferred direction of a cell or the number of head turns between trials. Both factors merit further investigation in future studies.

### 3.5 A Ring Attractor Network With Firing Rate Adaptation Accounts for Phase Precession Relative to Turning Angle

**FIGURE 6:**
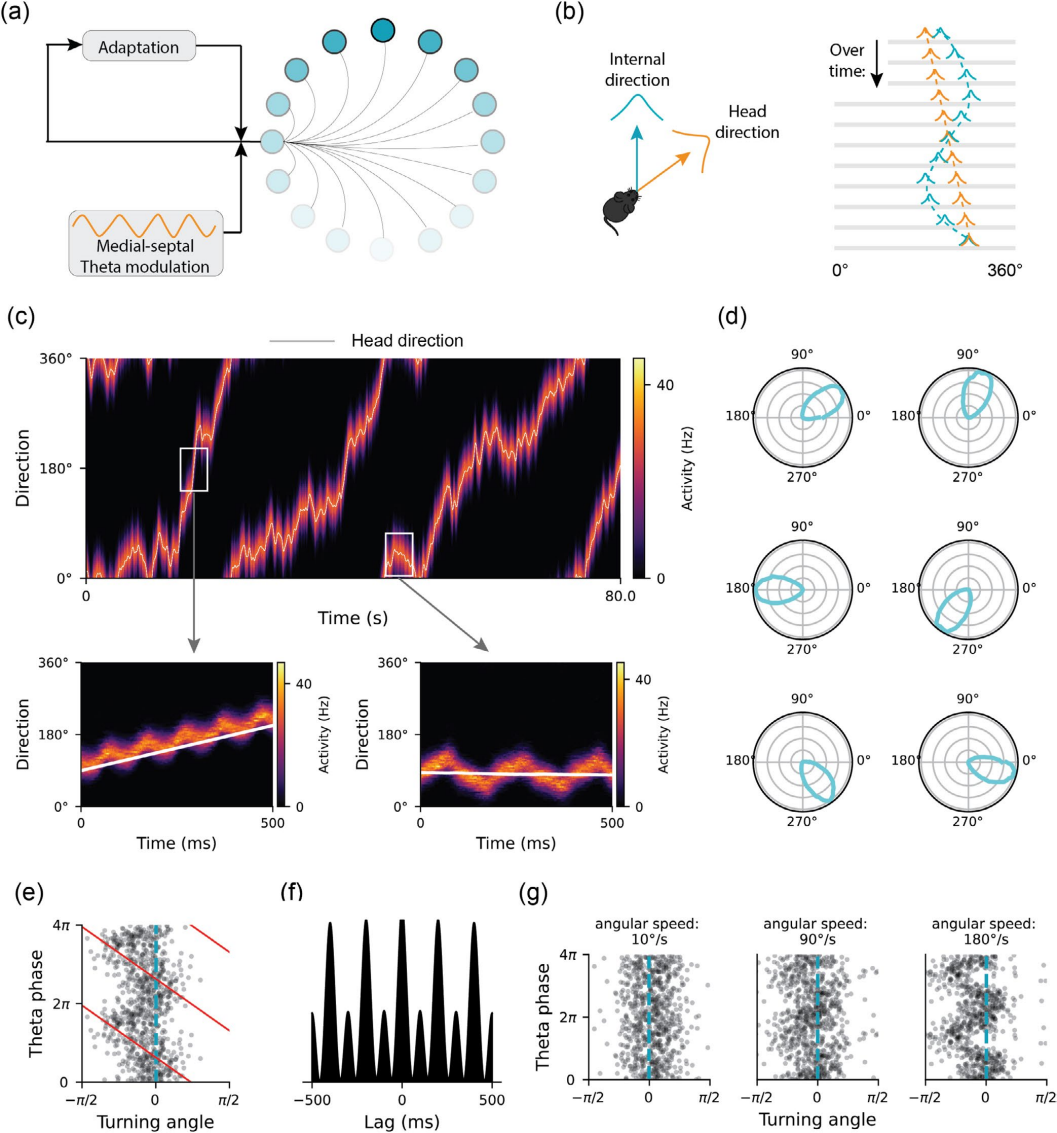
Theta phase precession relative to turning angle in a HD ring attractor model with firing rate adaptation. (a) The schematic of the ring attractor model. Blue‐gray dots mark HDCs, with more blue representing a higher firing rate. Each HDC receives a theta oscillation input as well as internal firing rate adaptation. (b) left: Demonstration of the animal's head direction (orange) and internal direction (blue) from the population activity in the ring attractor. Right: Sweeps of internal direction around the head direction in a theta cycle. (c) top: Directional theta sweeps over an 80‐s simulation duration, with gray lines representing the head direction. Bottom: Forward‐direction sweeps along the rotating direction when the angular head velocity is high (left) and bidirectional sweeps when the head direction is fixed. (d) Example HDCs in the ring attractor network after simulation. (e) Theta phase precession relative to the turning angle of a HDC during fast rotation in the ring attractor network, with the red line representing the circular–linear fit and the blue dashed line representing the peak preferred firing direction. (f) The temporal autocorrelogram during fixed head direction periods. (g) From left to right: Increased degree of phase precession with faster angular head velocity.

We recently developed a network model (Ji et al. 2025) to explain the alternating left–right theta sweeps in medial entorhinal grid cells (Vollan et al. 2024), including the role of tmHDCs in their generation. This latter part of the model consists of a ring attractor network with recurrently connected HDCs (Zhang 1996), modulated by an external theta rhythm (from medial septum and/or ventral tegmental nuclei of Gudden) and possessing internal firing rate adaptation (see Figure 6a and Methods for details). Previous work has shown that firing rate adaptation in attractor dynamics can lead to sweeps of the activity bump (see Figure 6b and Chu et al. (2024)). Here, we show that this model generalizes beyond grid and place cells, modeling the generation of phase precession relative to turning angle within the tmHDC ring attractor. As the simulated animal turns its head, the population activity closely tracks the head direction (Figure 6c) and cells in the network exhibit localized tuning curves tuned to different directions (Figure 6d).

Interestingly, depending on the angular speed, the population activity displayed either forward‐directed sweeps from the current to future head directions in each theta cycle or bidirectional sweeps from side to side of the head axis in alternate theta cycles (Figure 6c). Thus, when the animal turns its head, these cells fire at a late phase when head direction enters the preferred tuning field, progressing to earlier phases as head direction moves through the field, demonstrating phase precession relative to turning angle (Figure 6e). In contrast, when the animal runs in a straight line aligned with the cells' tuning fields, they fire every two cycles, displaying the theta‐skipping effect (Figure 6f). These results indicate that theta phase precession and theta skipping are single‐cell firing features of a shared population dynamic, contingent on whether the animal rotates its head or moves in a straight line. Furthermore, as the angular speed of head rotation increases, the degree of phase precession relative to turning angle also increases, distinguishing low‐speed from high‐speed turns in theta phase coding (Figures 3d and 6g).

### 3.6 HD Cells in the AVN Show Firing Rate Adaptation as the Head Continues to Point in the Preferred Direction

**FIGURE 7:**
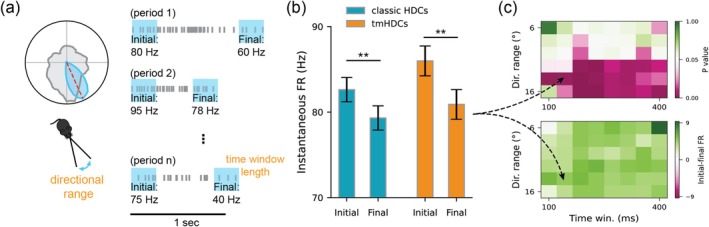
HD cells in the AVN show firing rate adaptation as the head continues to point in the preferred direction. (a) The schematic of the calculation of initial and final firing rates of HD cells during episodes when the animal continuously points its head within a range of the preferred direction of the cell for a duration of at least a specified time. (b) Mean instantaneous firing rates of both classic HDCs and tmHDCs during all initial episode periods and final episode periods, with error bars representing the standard error of the mean. **Wilcoxon sign rank test (WSRT) with p= 9×$10^{-5}$ in classic HDCs; **(WSRT) p=1.9×$10^{-3}$ in tmHDCs. (c) Top: *p* values of the condition effect (initial vs. final periods) from a two‐way ANOVA with respect to hyperparameters, including the range of directions around the preferred direction and the length of the initial/final periods. *p* values smaller than 0.05 are shown in pink, while those larger than 0.05 are shown in green. Bottom: Mean firing rate differences between the initial and final episode periods with respect to hyperparameters, with positive values shown in green and negative values shown in pink.

Our computational modeling suggests that HDCs must exhibit firing rate adaptation to display phase coding and theta‐skipping features. Here, we investigated whether HDCs in the AVN demonstrate firing rate adaptation using the method proposed in Taube and Muller (1998). Specifically, for each HD cell, we identified episodes in which the animal continuously pointed its head within $\pm 15^{{}^{\circ}}$ of the cell's preferred direction for at least 300 ms (Figure 7a). This resulted in 1754 episodes from 136 classic HDCs (12.9 episodes per cell) with a mean episode duration of 608 ± 15 ms and 1843 episodes from 223 tmHDCs (8.3 episodes per cell) with a mean episode duration of 593 ± 14 ms. In each episode, we calculated the mean instantaneous firing rate during the initial 150 ms and the final 150 ms.

We found that both classic HDCs and tmHDCs exhibited significantly higher firing rates during the initial episode period compared to the final episode period (Figure 7b; two‐way ANOVA with $F\left(1,7190\right)=6.98,p=0.008;$ classic HDCS: 82.6 ± 1.4 Hz vs. 79.3 ± 1.4 Hz; tmHDCS: 86.0 ± 1.7 Hz vs. 80.1 ± 1.7 Hz). Furthermore, this firing rate difference was robust to variations in the analysis parameters, including the range of directions around the preferred direction ($\pm 6^{{}^{\circ}}$ to $\pm 16^{{}^{\circ}}$) and the duration of the initial and final periods (100–400 ms) (Figure 7c). The *p* values were less than 0.05 for many parameter combinations (27 out of 48), and the firing rate consistently remained higher during the initial periods than the final periods.

Additionally, there is no evidence of a difference in firing rates during the initial and final periods between the two cell groups (two‐way ANOVA with $F\left(1,7190\right)=2.396,p=0.122$). Furthermore, there is no evidence that tmHDCs exhibit a stronger or weaker adaptation effect compared to classic HDCs, as indicated by the comparison of the differences between their initial and final episode periods (two‐sample *t*‐test with t=0.685,p=0.493).

## 4 Discussion

We have provided evidence of theta phase coding relative to turning angle in the tmHDCs in the anteroventral thalamus (Tsanov et al. 2011; Lomi et al. 2023). These findings broaden our understanding of neural phase coding in spatial orientation and navigation beyond the phase coding of distance seen in the hippocampal formation and interconnected regions. Additionally, we show that a ring attractor network model of tmHDCs with firing rate adaptation (see also Ji et al. 2025 for more details), which explains the generation of theta sweeps of internal head direction. This model accounts for the observed single‐cell firing features—phase precession relative to turning angle and theta skipping (firing on alternate theta cycles)—providing a unified framework for understanding information coding by tmHDCs, as well as in the entorhinal‐hippocampal spatial navigation system see Robinson and Brandon (2021) and the extended model presented in Ji et al. (2025).

The anterior thalamus is considered crucial to several interdependent diencephalic‐hippocampal circuits that process theta rhythmicity and spatial information. Within the anterior thalamus, the ventral and dorsal nuclei (AVN; ADN) can be anatomically and functionally dissociated, forming separate systems that differently contribute to limbic functions (Aggleton et al. 2010). By virtue of its anatomical and electrophysiological properties, the ADN supports the classic HDCs system conveying global directional inputs to the hippocampal formation. On the other hand, the AVN is part of a theta system that (together with the medial septum) propagates theta activity to the hippocampal formation (Jankowski et al. 2013), characterized by the presence of theta‐modulated cells at all levels of the circuitry (Bassant and Poindessous‐Jazat 2001; Kocsis et al. 2001; Vertes et al. 2001; Albo et al. 2003; Sharp and Turner‐Williams 2005). By comparison, only small numbers of theta cells are found along the classic HDC system (Vertes et al. 2001; Albo et al. 2003). These two pathways ascend to the hippocampus with a parallel organization, respectively, supporting head direction vs. theta processing, as follows: tegmental nuclei of Gudden (dorsal vs. ventral; DTN vs. VTN), mammillary bodies (lateral vs. medial; LMN vs. MMN), anterior thalamic nuclei (dorsal vs. ventral; ADN vs. AVN), retrosplenial/subicular/entorhinal cortices, and finally the hippocampus (Witter et al. 1990; Shibata 1993; Van Groen and Wyss 1995; Van Strien et al. 2009; Shibata and Yoshiko 2015; Christiansen et al. 2016; Lomi et al. 2021). The AVN provides a functional crossover between these two pathways given the presence of tmHDCs, which could arise from converging MMN theta input and a corticothalamic HDC input, in turn inherited from the ADN.

In addition to the MMN input, the AVN is influenced by a strong descending hippocampal input via the subiculum (Seki and Zyo 1984; Mathiasen et al. 2019). Moreover, the MMN receives converging inputs from the medial septum and VTN (Shibata 1989), both being implicated in modulating hippocampal theta (King et al. 1998; Vertes et al. 2004). In this sense, the AVN receives and propagates two types of theta signals: one mainly descending from the septo‐hippocampal system and another ascending from brainstem regions (Kocsis et al. 2001). This could explain why medial septum inactivation does not always eliminate theta rhythmicity in parahippocampal regions and why theta oscillation in the retrosplenial cortex can persist or increase in the absence of hippocampal theta (Young and McNaughton 2009) or after electrolytic medial septum lesions (Borst et al. 1987). Given this influential contribution of the AVN to theta processing, it is noteworthy that theta‐burst optogenetic stimulation of AVN glutamatergic neurons restores spatial working memory performance in rats after mammillothalamic tract lesions and increases theta activity and immediate early gene expression within hippocampal formation structures (Barnett et al. 2021).

**FIGURE 8:**
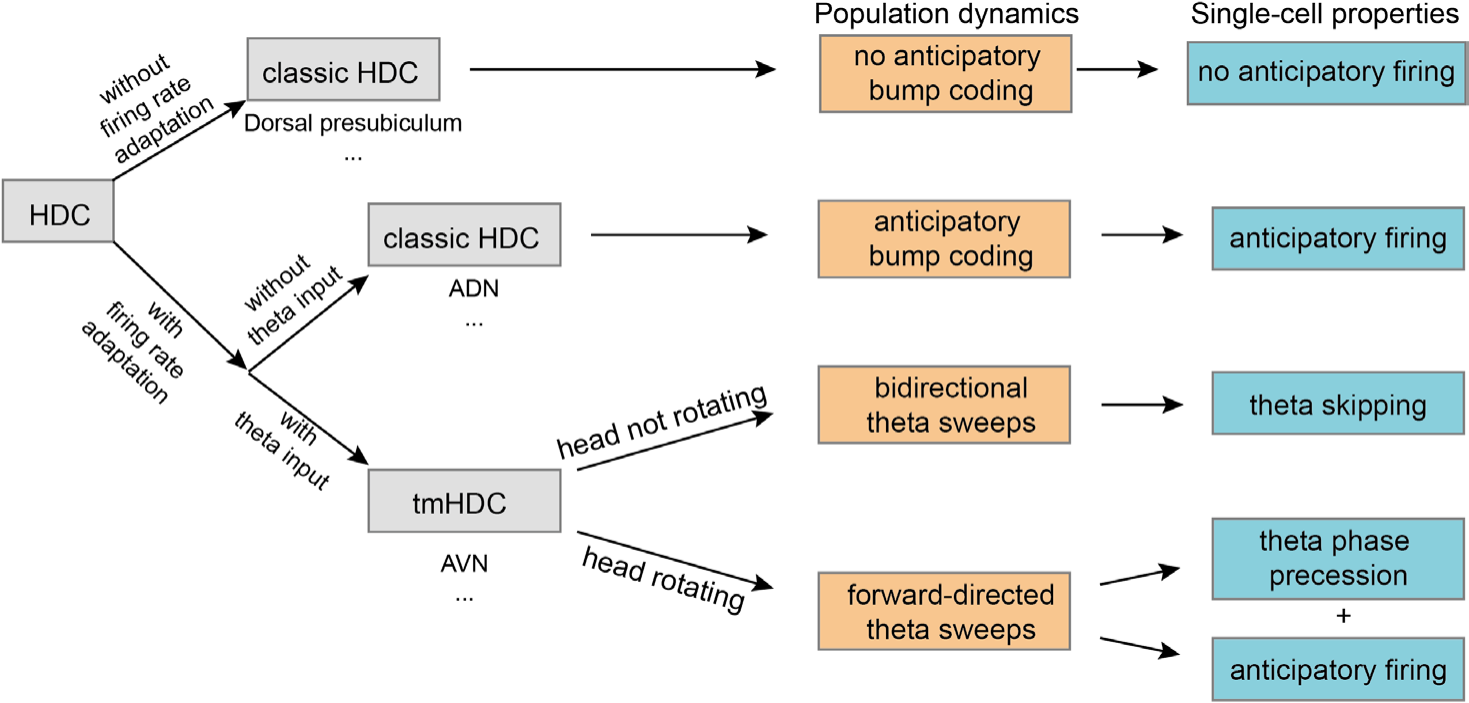
A model‐inspired diagram illustrating the classification of tuning features in head direction cells. The diagram shows that anticipatory firing, theta skipping, and phase precession are distinct single‐cell firing features arising from a shared population activity dynamic within the head direction attractor network. These features depend on whether the cells exhibit internal firing rate adaptation and receive external theta input.

Many single‐cell level phenomena in HDCs can be explained by population dynamics within the head direction network (Figure 8). First, during turning, the activity bump (representing the “internal head direction”) exhibits forward‐directed theta sweeps along the rotation direction (Figure 6c). Consequently, individual cells exhibit theta phase precession during head rotations through the preferred firing direction (Figures 2 and 5e), similar to place cells when the animal is traversing through the firing fields. Interestingly, higher angular head velocity leads to more significant forward‐directed sweeps in the modeled network. This results in an increased degree of phase precession during faster head turns, both in the model (Figure 6g) and the experimental data (Figure 3d). Second, during straight runs without head turning, the activity bump exhibits bidirectional theta sweeps around the animal's heading axis (Figure 6c). This phenomenon results in a theta‐skipping effect, where tmHDCs fire in alternative theta cycles (Figure 6f), and is consistent with theta‐skipping HDCs in MEC and parasubiculum that fire on the same cycles having more similar preferred firing directions than those that fire on opposite cycles (Brandon et al. 2013). Third, as the animal turns, forward‐directed sweeps result in anticipatory coding where the averaged center of the internal head direction moves ahead of the actual head direction. This time‐averaged effect leads to anticipatory firing of tmHDCs, as observed by Lomi et al. (2023). Although anticipatory firing has been reported previously in classic HDCs (Blair and Sharp 1995; Taube and Muller 1998; Lozano et al. 2017), and also modeled in a ring attractor network with firing rate adaptation (Mi et al. 2014), anticipatory firing in tmHDCs are likely a result of forward‐directed sweeps during head turning periods.

Previous calculation of cell's theta‐skipping indices was based on the autocorrelogram calculated from the spike train across the whole recording session (Brandon et al. 2013; Lomi et al. 2023). However, the specific amount of theta skipping in a single cell depends on the behavioral state and directional sampling of the animal: specifically, the amount of time spent facing the cell's preferred firing direction and the number of head‐turning movements. Our model suggested that theta skipping is more pronounced when the head is not rotating. Therefore, filtering out those rotating periods and using the remaining spike train to calculate the autocorrelogram can lead to a more accurate calculation of the theta‐skipping index. When we do this, the correlation between the degree of phase precession and the theta‐skipping index in Figure 4a improves. Overall, theta phase precession relative to turning angle and theta skipping are two single‐cell firing features of population activity sweeps within the tmHDCs network, whose occurrence depends on whether the animal rotates its head (one leads to forward‐directed sweeps and the other leads to bidirectional sweeps; see Figure 7). This aligns with the experimental finding of a higher proportion of phase‐precessing cells among theta‐skipping HDCs (Figure 3e), as well as an increased degree of phase precession with the theta‐skipping index (Figure 4a).

A key feature for modeling phase precession and theta‐skipping phenomena in the ring attractor network is that cells undergo theta modulation and firing rate adaptation (Figure 6). Previous work showed that HDCs in the dorsal presubiculum do not exhibit firing rate adaptation (Taube and Muller 1998), a finding further supported by evidence of persistent firing driven by intrinsic mechanisms in dorsal presubiculum neurons observed in in vitro whole‐cell patch recordings (Yoshida and Hasselmo 2009). In contrast, whether HD cells in the ADN exhibit firing rate adaptation remains less clear, as they were reported to show significant firing rate adaptation during session 1 but not session 2 (Taube and Muller 1998). Given that firing rate adaptation in a ring attractor model leads to anticipatory firing of HDCs (Mi et al. 2014)—a phenomenon observed in the ADN but not in the dorsal presubiculum—it is likely that ADN HDCs also exhibit firing rate adaptation. Using the same analysis, we provided the first evidence of firing rate adaptation in AVN HDCs, consistent with the anticipatory firing observed in AVN HDCs (Lomi et al. 2023), The significance of this finding, however, depends on the hyperparameters used in the analysis (Figure 7c). For example, when using the same parameters as (Taube and Muller 1998) (directional range: $\pm 6^{{}^{\circ}}$, window size: 150 ms), the effect of firing rate adaptation is sometimes not significant. Nevertheless, across different parameter choices, the trend of higher firing rates during the initial period remains robust and independent of specific parameters. Importantly, firing rate adaptation was measured exclusively during head‐stationary periods, whereas phase precession was analyzed only during head rotation periods. This distinction highlights that one neural feature (firing rate adaptation) during a specific behavioral state (forward movement of the head) predicts another neural feature (angular phase coding) during a different behavioral state (rotation of the head). As mentioned above, a possible pathway of directional sweeps in the brain could arise from direct AVN projections to postsubiculum, parasubiculum, and medial entorhinal cortex. These regions contain tmHDCs that can exhibit theta skipping (Brandon et al. 2013), suggesting an involvement in phase precession and therefore in forward theta sweeps of location. Cortical tmHDCs can also show a stronger spatial component, in the form of grid‐by‐HDCs in the medial entorhinal cortex (Brandon et al. 2013) and place‐by‐HDCs in the post‐ and parasubiculum (Cacucci et al. 2004). These more conjunctive representations could provide the circuit level mechanisms to mediate the bidirectional theta sweep dynamics on alternating cycles shared across the representations of direction and location (Johnson and Redish 2007; Kay et al. 2020; Vollan et al. 2024; Widloski and Foster 2024). Moreover, they provide an efficient mechanism for scanning locations in the ambient environment during navigation (Erdem and Hasselmo (2012); Vollan et al. (2024); Ji et al. (2025).

Future work to further explore the contribution of these single‐cell firing dynamics to hippocampal‐related memory processes could include simultaneous recordings from the hippocampal formation during navigation and sleep, coupled with AVN inactivation. It will be particularly informative to record in tasks that favor head rotations to maximize the opportunity to observe angular phase precession, for instance, using a repetitive circle task (Brandon et al. 2011) or in virtual reality. Finally, given that hippocampal theta sequences are related to offline replay in location, in both developmental data (Muessig et al. 2019) and disruption studies (Drieu et al. 2018), it would be interesting to investigate the relationship between phase precession in the directional domain and replay in location. For example, models of replay via Hebbian strengthening of connections between place and head direction information (Hasselmo 2008; Hasselmo and Brandon 2008) might apply better to tmHDCs (which are involved in theta sweeps) than classic HDCs (which do not replay spatial trajectories during REM sleep, see Brandon, Bogaard et al. (2011), but also see Peyrache et al. (2015).

## 5 Conclusions

Our study provides evidence of theta phase coding relative to turning angle in tmHDCs, extending the concept of theta phase coding beyond the spatial dimension observed in grid and place cells. This finding underscores the versatility of theta phase coding mechanisms in encoding both spatial and directional information, potentially supporting more complex navigation strategies that integrate head orientation and movement trajectories. Additionally, we relate the population‐level phenomenon of theta sweeps within the HD attractor network to three fundamental aspects of single‐cell firing in HDCs, providing a unified framework to explain these apparently distinct single‐cell findings: first, tmHDCs in the AVN show a greater degree of phase precession during faster head turns; second, tmHDCs involved in phase precession during head turns are also more likely to display theta skipping during stationary head periods; third, tmHDCs in the anterior thalamic nucleus exhibit increased anticipatory firing (Lomi et al. 2023), a time‐averaged effect of forward‐directed theta sweeps. These findings also relate to the generation of left–right sweeps in grid cells and place cells (see Vollan et al. (2024) and Ji et al. (2025)). Finally, we provide the first evidence of firing rate adaptation in HDCs in the AVN, which supports the computational model unifying these findings by linking firing rate adaptation to theta phase coding, theta skipping, and anticipatory firing.

## Author Contributions

Zilong Ji and Neil Burgess conceptualized the project. Eleonora Lomi conducted all experiments with input from Kate Jeffery and Anna S. Mitchell. Zilong Ji and Eleonora Lomi analyzed the experimental data with input from Neil Burgess. Zilong Ji built the computational model and performed the simulations. All authors discussed and interpreted the results. Zilong Ji, Eleonora Lomi, and Neil Burgess wrote the first draft of the paper. All authors edited the final manuscript draft.

## Conflicts of Interest

K.J. is a non‐shareholding director of Axona Ltd.

## Data Availability

The dataset has been presented in Lomi et al. (2023) and is available at: https://figshare.com/articles/dataset/_strong_Data_code_for_Lomi_et_al_2023_strong_/22802861. The code for reproducing all the results (including modeling and experimental data analysis) is available at: https://github.com/ZilongJi/HDPhasePrecession.
